# Application of computer-aided design and 3D-printed template for accurate bone augmentation in the aesthetic region of anterior teeth

**DOI:** 10.1186/s12903-023-02707-7

**Published:** 2023-01-10

**Authors:** Xin Liu, Zhou Fang, Jin Feng, Se-fei Yang, Yi-peng Ren

**Affiliations:** 1 Department of Oral and Maxillofacial Surgery, Chinese PLA General Hospital, Beijing, 100853 China; 2 Air force medical center of Chinese PLA, Beijing, 100000 China

**Keywords:** Virtual surgery, Implant restoration, Aesthetic zone, Bone augmentation

## Abstract

**Purpose:**

To explore the outcomes of bone augmentation in the aesthetic zone of the anterior teeth using computer-aided design and a 3D-printed template.

**Methods:**

Ten patients with severe bone defects in the aesthetic zone of anterior teeth were included in the study; CT data were collected before surgery. The design of the osteotomy line in the bone defect area was determined under computer simulation. The position parameters and osteotomy line of the free bone were determined via virtual surgery. A 3D-printed template was prepared to guide the accurate placement of the bone graft. Reexamination was conducted to evaluate the position of the bone graft immediately after the operation and the resorbed capacity of the bone graft before implant restoration.

**Results:**

The position of the bone graft was consistent with the preoperative design. The amount of bone graft resorbed was within the acceptable range three months after the operation, and the effect of implant restoration was satisfactory.

**Clinical significance:**

Use of computer-aided design and a 3D-printed template can be an effective approach for accurate bone augmentation in the aesthetic zone of the anterior teeth.

Implant restoration in the aesthetic zone of the anterior teeth should be performed considering the aesthetic effect of the proportion of the pink (gingiva) and white (teeth) regions, in addition to ensuring that the biting and cutting functions are maintained [1, 2]. This has always been challenging in clinical implant restorations, especially in cases of severe bone loss; further, the treatment options for such cases are limited [3, 4]. At present, the main treatment for severe bone defects is onlay bone grafting with autologous bone. There are no specific criteria for determining the amount of bone graft, location of bone graft, appropriate treatment for the bone defect area, design of donor bone morphology, and postoperative prediction of free bone resorption[5]. The surgical operation for treatment of such bone defects is not standardized [5]. With continued developments in computer-aided design and 3D-printed technology, it has become widely used in the field of stomatology [6, 7]. It is characterized by high accuracy, and is useful for performing oral implantation, which includes the use of 3D-printed implants and guide plates [8]. However, its application in bone augmentation surgery in the aesthetic zone of the anterior teeth has not yet been explored. Therefore, this study was performed to obtain a precise design for bone graft placement in the aesthetic zone of the anterior teeth using computer-aided design. On this basis, we created a 3D-printed template, performed bone grafting, and completed implant restoration to achieve precise bone augmentation to ensure optimal outcomes of implant restoration.

## Cases and methods

### Patient selection

Ten patients (seven females and three males) were selected from the Department of Dental Implantation of the Chinese PLA General Hospital from June 2014 to June 2017. These patients comprised four cases of periodontal disease-induced tooth loss, three cases of traumatic tooth loss, and three cases of tooth loss after local tumor resection. All patients had severe bone defects in the aesthetic zone of the anterior teeth; the duration of the defect was ≥ 6 months. Inclusion criteria were as follows: missing teeth ≥ 3; maximum vertical defect of alveolar ridge ≥ 1.5 cm; and no history of treatment with fixed denture restoration. Basic information of the patients is presented in Table 1.

**Table 1:** General characteristics of the patients

	Sex		Age (years)	Positions of the missing teeth	Duration of the defect (months)
1	Female		47	A1, B1, B2	6
2	Female		29	A2, A1, B1, B2	7
3	Male		31	A2, A1, B1, B2	13
4	Female		35	A1, B1, B2, B3	7
5	Female		42	A2, A1, B1	10
6	Female		46	A2, A1, B1	30
7	Female		31	A2, A1, B1, B2	12
8	Male		44	A2, A1, B1	29
9	Male		39	A1, B1, B2	8
10	Female		37	A1, B1, B2	31

### Computer-aided determination of osteotomy range in the bone defect area

A CT scan of the maxillofacial region was performed before surgery. Original DICOM data were collected. CT data of the jaw were segmented using ProPlan CMF 2 (Materialise). The maxilla was segmented separately, and a 3D reconstruction model was established. The design of the osteotomy line, which could remove the weak bone wall and define the shape of the bone defect area, was determined. The objectives for virtual osteotomy are as follows: (1) retaining the height of the interalveolar septa of the adjacent teeth, while maintaining a distance of ≥ 1.5 mm from the adjacent periodontal membrane; (2) trimming alveolar ridge crests with flat or inclined planes in the recipient site, removing uneven bone crests, and retaining a bucco-lingual width ≥ 5 mm in the alveolar bone; (3) ensuring a rectangular or trapezoidal osteotomy area, with no undercut, which does not hinder the placement of the bone graft.

### Computer-aided determination of the morphology of the free bone

**Fig. 1:**
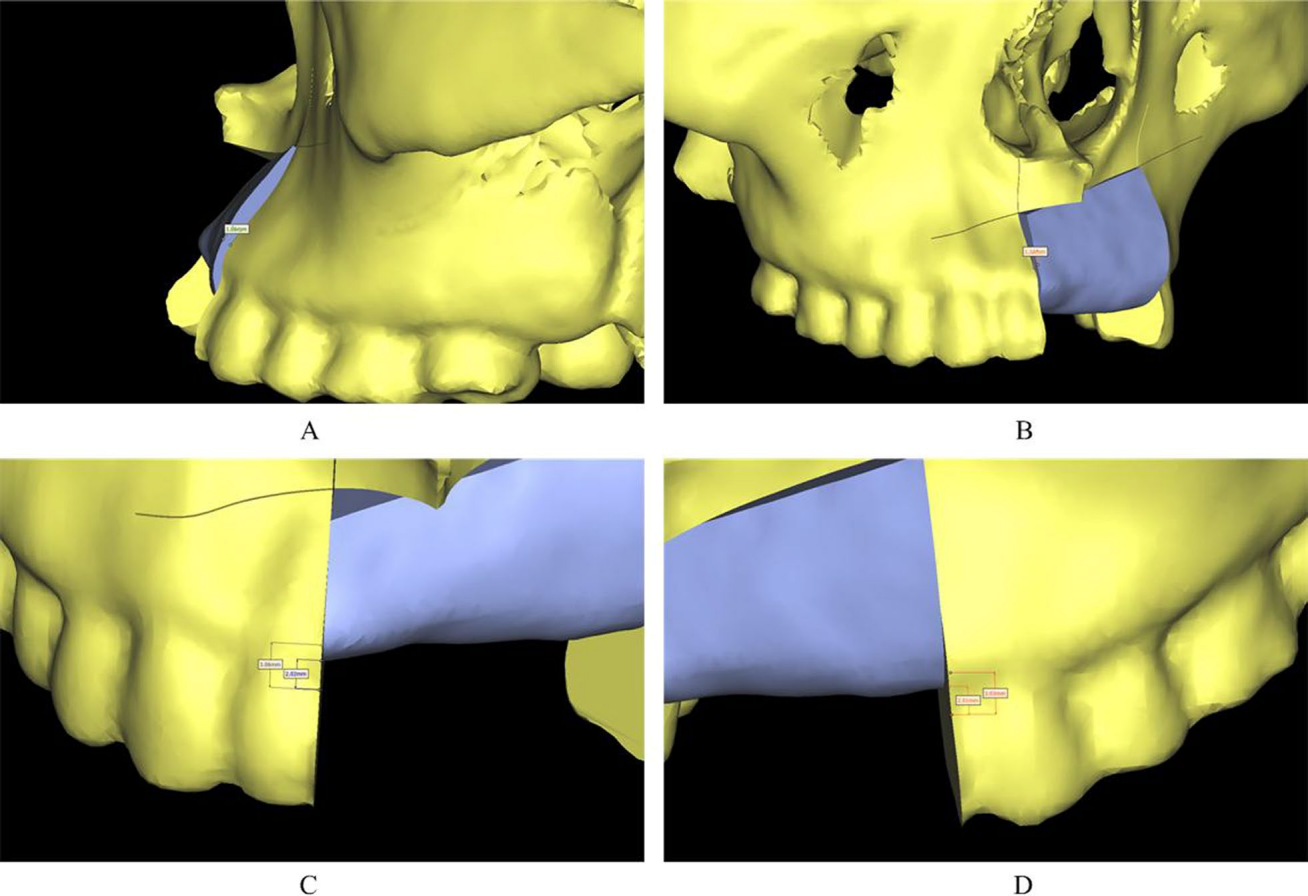
Design parameters: **A**, **B** the labial and palatine sides of the bone graft are 1 mm higher than the alveolar bone on both sides to accommodate for the expected bone resorption; **C**, **D** the maxillary and mandibular planes of the bone graft are located 1 mm below the enamel-cementum junction

CT data of iliac bone area were collected before surgery. The method described in the previous section was used to segment bone data and establish the 3D reconstruction model. The iliac bone model was superimposed on the model created for performing virtual maxillary osteotomy for determining the positional parameters and virtual osteotomy line. The design principles are as follows: (1) The pelvic side of the iliac crest moved toward the palatine side, and the iliac crest faced to the maxillofacial surface to make use of the radian of the anterior superior iliac crest; (2) the horizontal position of the free bone should be located 1 mm below the cemento-enamel junction of the adjacent tooth (2 mm at normal position; 1 mm of reservation for bone resorption); (3) the labial and palatine sides of the free bone should be 1 mm higher than the contour of the adjacent alveolar crest as the bone resorption margin; (4) the lateral cortical bone of the iliac crest is preserved as a labial surface (Fig. 1).

### 3D-printed template

(1) According to the determined location of the osteotomy line of the alveolar bone and iliac bone, the virtual osteotomy was performed by data segmentation. (2) The maxillary model after osteotomy and the 3D model of the iliac bone were established. (3) Data were loaded onto the forward engineering software 3-matic (Materialise NV, Leuven, Belgium). The osteotomy guide plates of the alveolar bone and the iliac bone were constructed separately. The placement guide plate was designed in accordance with the 3D model of the jaw after the iliac bone model was in place. (4) The resin model and guide plate were created using 3D printing.

### Surgical procedure and implant restoration

The surgery was performed based on computer-aided design technique. During the surgery, mini titanium plates and titanium nails (AO mini) were used to firmly fix the free bone. Four months after surgery, the titanium plates and titanium nails were removed, and the implant was placed. The submerged implant method, with the implant tilted to the palatine side, was used to ensure adequate bone thickness on the labial side after partial resorption of the donor bone. Three months later, a healing abutment was inserted during second-stage surgery. Three to four weeks later, the model was constructed and fixation was performed. For some patients with high aesthetic requirements, temporary denture restoration was performed after obtaining the model; permanent restoration was carried out 3 to 6 months after gingival shaping.

### Postoperative verification

The following two aspects are the main considerations for postoperative verification: (1) Evaluation of accuracy of bone position: maxillofacial CT data reexamined within 2 weeks after the surgery and preoperative design data were loaded onto reverse engineering software geomagic control. Preoperative and postoperative image matching was conducted, and the reconstruction accuracy was analyzed. (2) Evaluation of effect of bone augmentation: a CT scan was performed 4 months after surgery to analyze the amount of bone resorption in each direction and to assess the effect of bone augmentation.

### Statistical analysis

Data were analyzed with independent samples *t*-test using SPSS 17.0 software (SPSS Inc., Chicago, IL).

## Results

### Accuracy of bone position

The average position deviation between the postoperative bone location and preoperative design position was 0.45 ± 0.37 mm.

### Effect of bone augmentation at 4 months postoperatively

CT data immediately after the surgery and 4 months postoperatively demonstrated that (1) mean vertical bone absorption of the free bone was 1.09 ± 0.14 mm. (2) Mean absorption of free bone mass was 1.24 ± 0.24 mm on the labial side and 1.09 ± 0.28 mm on the palatal side. (3) The bone contact surface was well healed. (4) No significant bone resorption was found in the adjacent alveolar bone (Table 2).

**Table 2:** Measurement indicators and statistical results (*P < 0.05)

Resorption of bone graft (mm)	Mean ± standard deviation	Target value	*P*
Vertical direction	1.09 ± 0.14	1	0.068
Palatine side	1.09 ± 0.28	1	0.377
Labial side	1.24 ± 0.24	1	0.011*

*P values are obtained from *t*- test; significant difference, *P* < 0.05

### Typical case

**Fig. 2:**
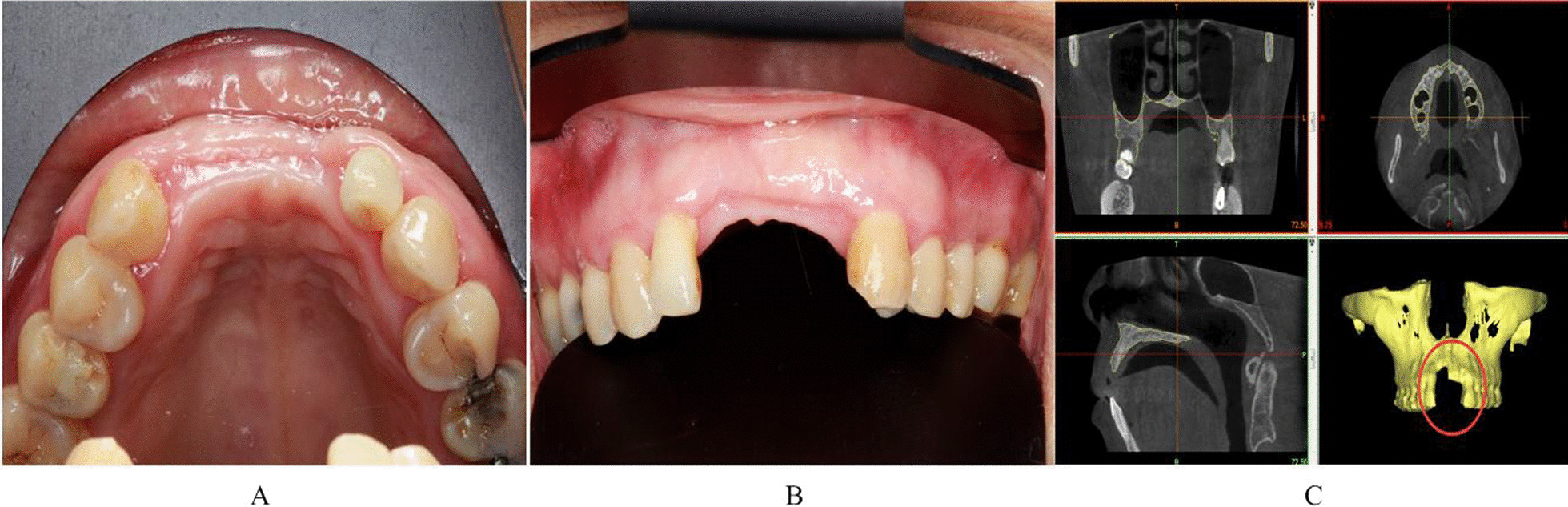
Preoperative intraoral photograph: **A** maxillofacial view; **B** front view; **C** CT imaging and 3D reconstruction

**Fig. 3:**
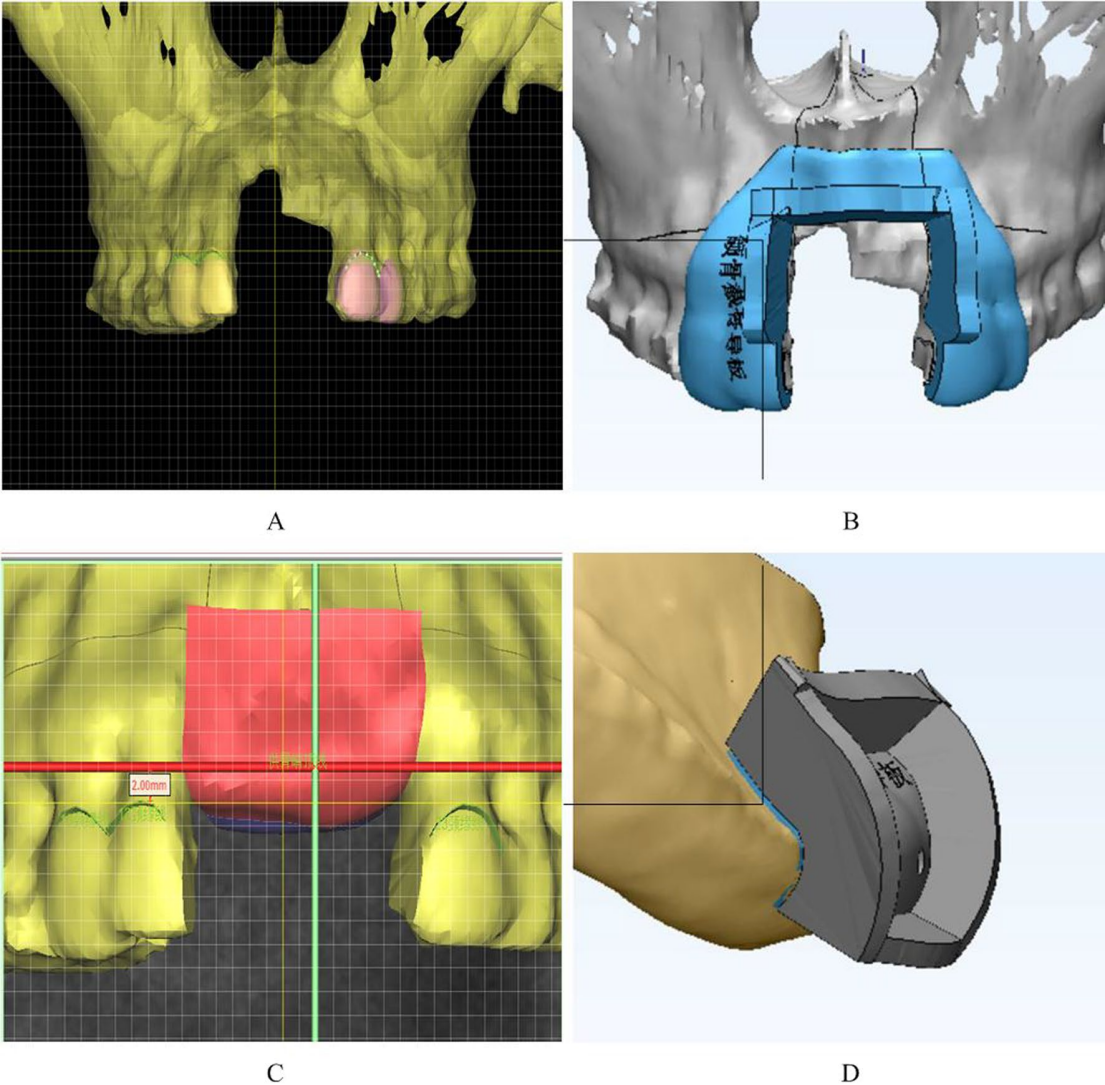
Computer-aided design: **A** identifying the enamel-cementum junction of the adjacent tooth; **B** determining the osteotomy design in the bone defect area; **C** design of the bone graft location; **D** iliac osteotomy design

**Fig. 4:**
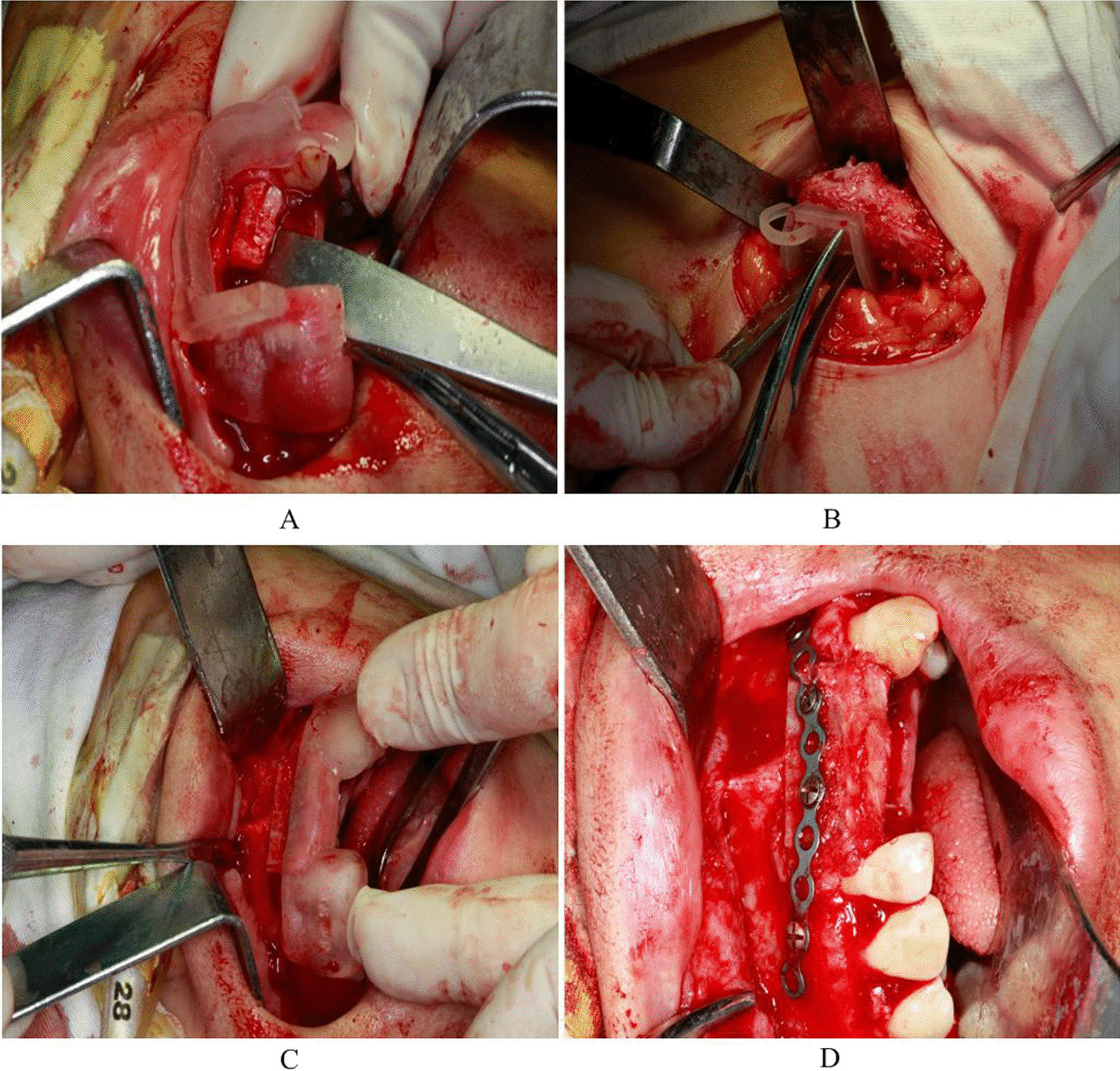
Implementation of template-assisted surgery: **A** placement of the guide plate and osteotomy at the surgical area; **B** iliac osteotomy guide plate design and bone cutting; **C** design of the bone placement guide plate and placement of the bone; **D** bone fixation

**Fig. 5:**
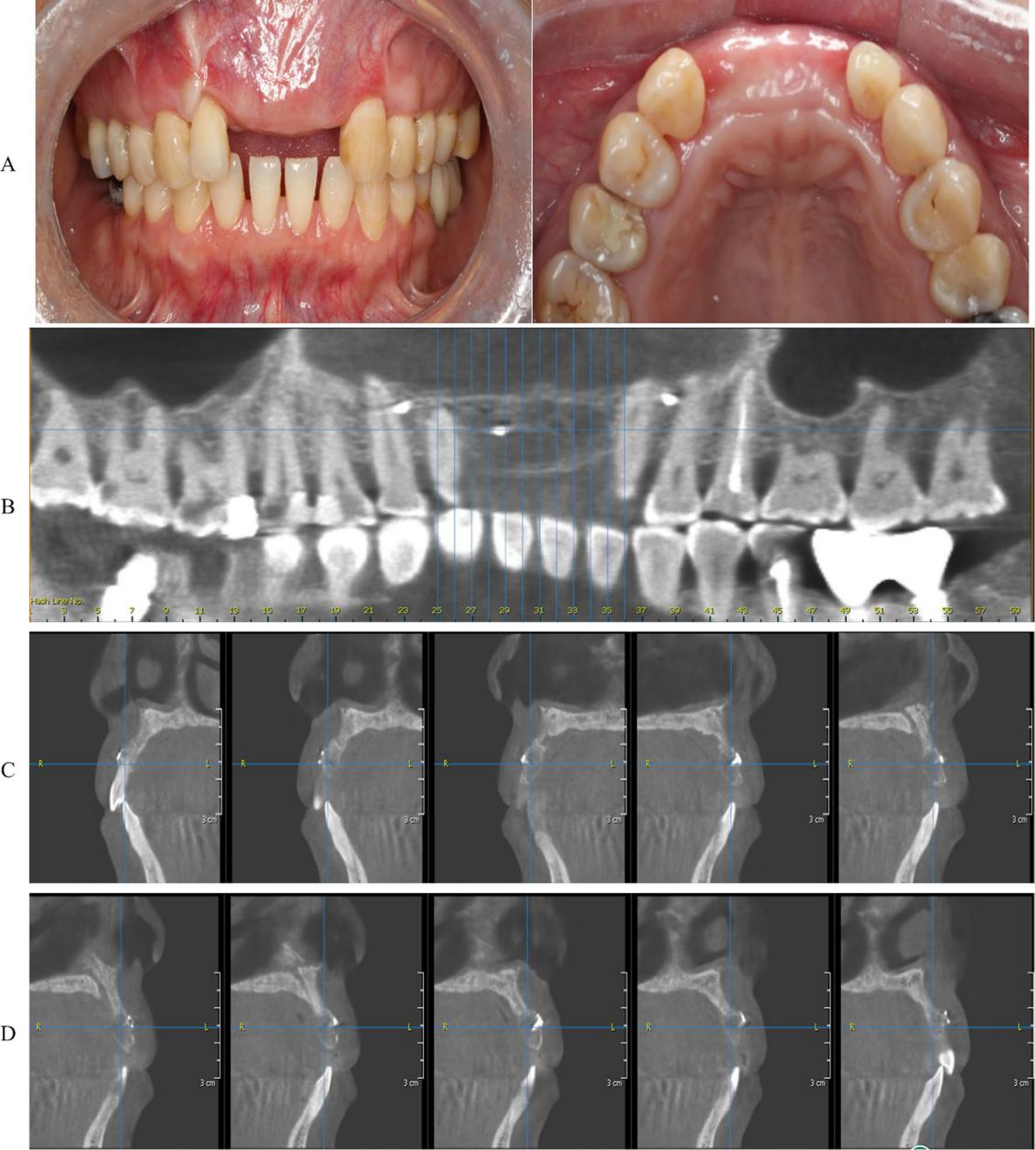
Healing and bone resorption revealed by cone beam CT at 4 months postoperatively: **A** intraoral photo; **B** bone graft position; **C**, **D** cone beam CT shows local bone resorption

**Fig. 6:**
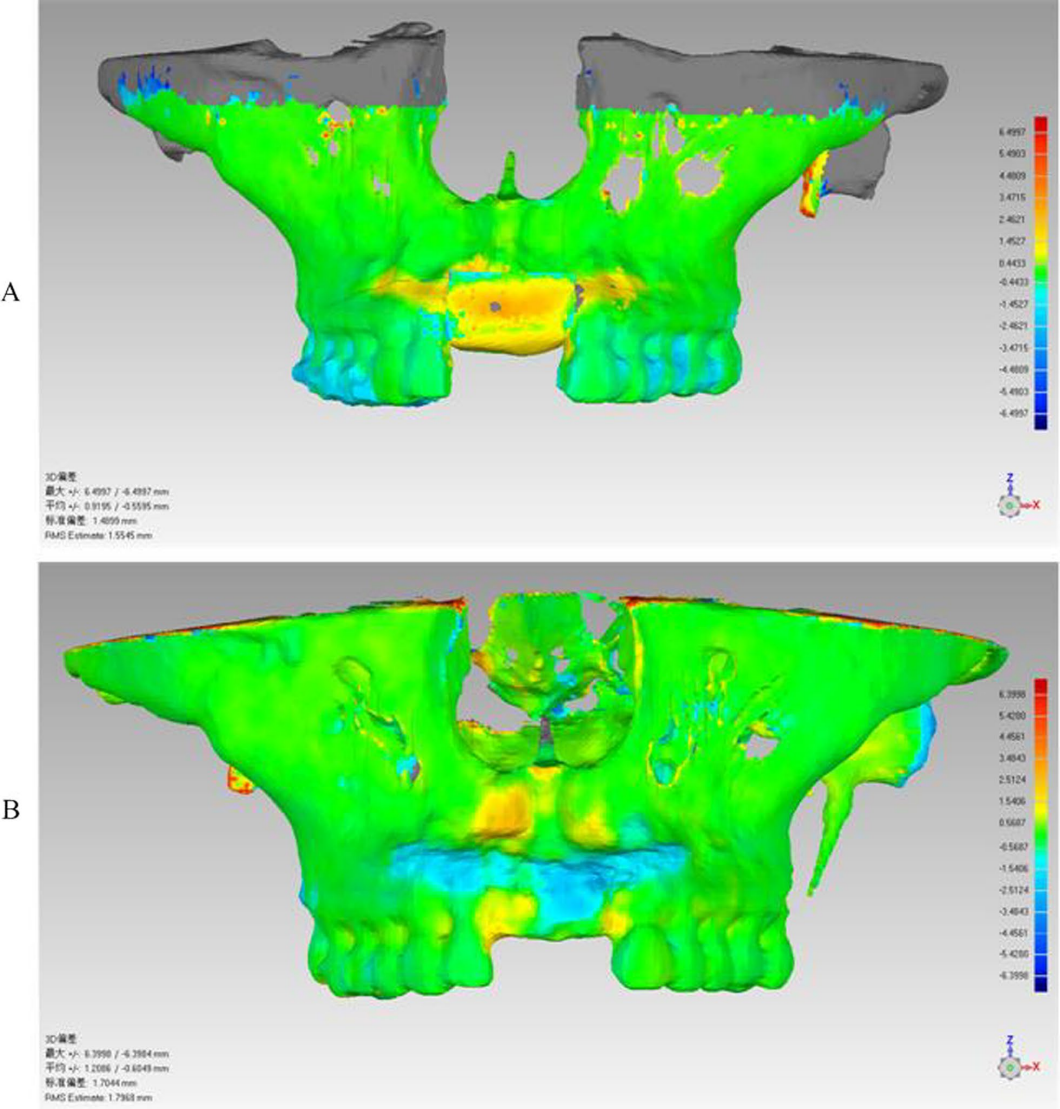
Color temperature chart of bone graft location: **A** comparison of CT results preoperatively and 2 weeks postoperatively, showing that the average error of bone position accuracy is 0.73 mm; **B** comparison of CT results between 2 weeks and 4 months postoperatively, showing bone resorption on the labial and alveolar ridge crest, especially on the labial side

**Fig. 7:**
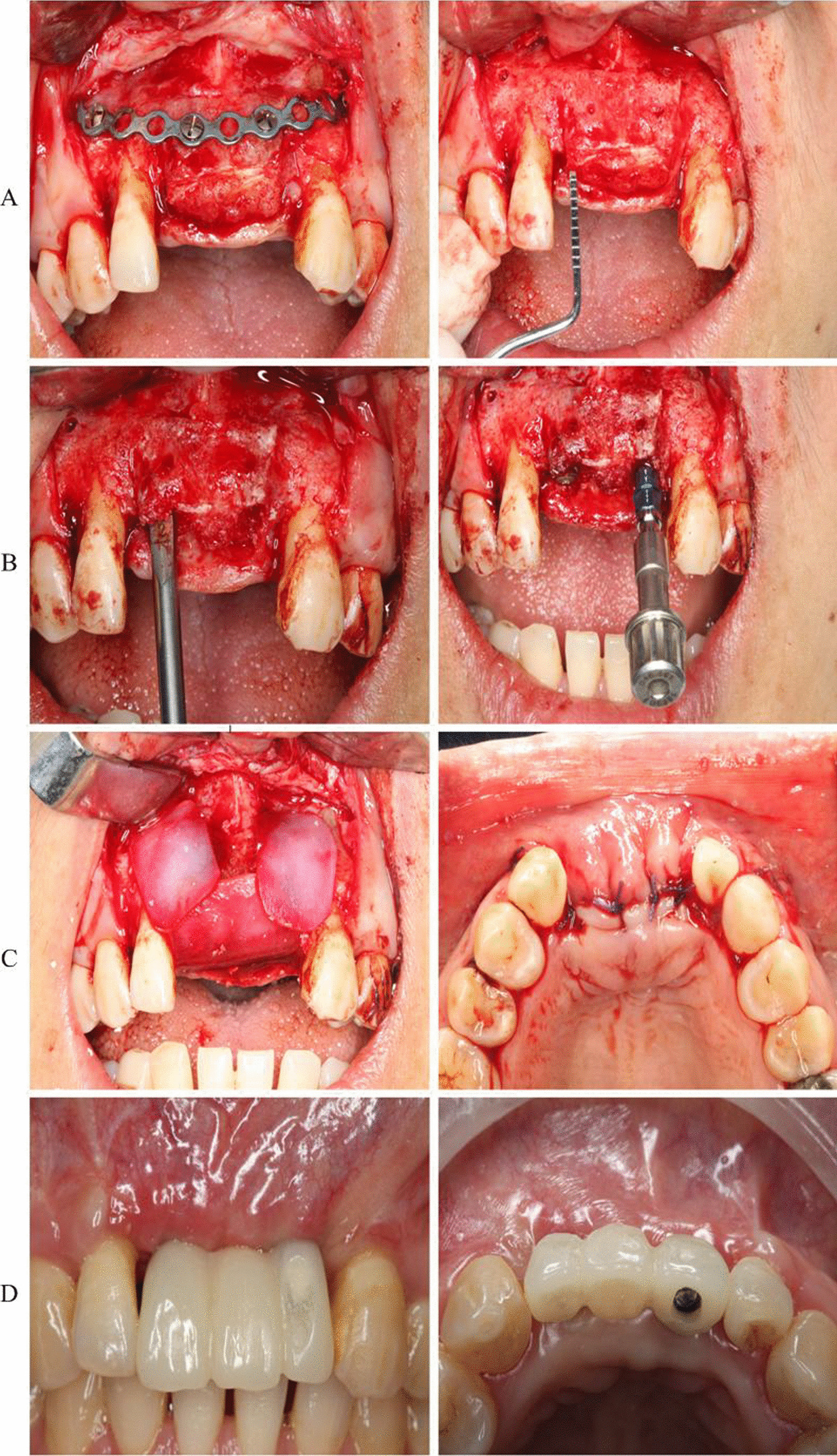
Implant restoration: **A** Removal of titanium plate and assessment of bone resorption at 4 months postoperatively; **B** bone splitting and implant placement; **C** covering a bioabsorbable membrane (Bio-Gide, Geistlich, Switzerland) and suturing the incision; **D** Restoration completion

A 52-year-old female patient developed local infection caused by apical inflammation of the right maxillary central incisor. After removing the affected tooth, local bone resorption was found to be extensive. She visited our hospital in December 2016. With the aid of computer-aided design and the 3D-printed template, a free iliac bone graft was used to repair the local bone defect and implant restoration was performed (Figs. 2, 3, 4, 5, 6 and 7).

## Discussion

The aesthetic region of the anterior teeth comprises the anterior teeth and surrounding tissues that are exposed when smiling. In general, it includes the regions of the incisors, canines, and premolars[9–11]. Defects in the anterior teeth not only affect normal oral function, but also affect the beauty of the patient’s face, which has a negative impact on the patient’s psychology[12]. A removable denture may be used for the restoration of such defects, but it has certain shortcomings, such as inadequate functional recovery, poor appearance, sensation of a foreign body in the mouth, and inconvenience associated with the frequent insertion and removal of the prosthesis[13]. In contrast, a fixed denture can cause wear of the healthy adjacent teeth, cause fracture or mobility of the abutment teeth, and cause symptoms of dental pulp inflammation. Therefore, fixed dentures often cause considerable pain in the patients[13–15]. For continuous loss of multiple teeth, as the span of the bridge is large, it is difficult to use all-porcelain restoration[12, 16]. Therefore, implant restoration remains the optimal method for restoration in the aesthetic region of the anterior teeth[16, 17]. The shape of an implant restoration is lifelike and provides favorable aesthetic results; further, the restoration is stable, comfortable, and hygienic[18]. In particular, excellent recovery of chewing function is observed, and hence, implant restorations are widely accepted by patients.

Nevertheless, in clinical practice, a considerable number of patients have bone defects in the aesthetic region of the anterior teeth and require treatment with bone augmentation. In some cases, bone augmentation can be achieved through guided bone regeneration and bone splitting. In some cases with large bone defects, onlay bone grafting and distraction osteogenesis can be performed to overcome the problem of insufficient bone mass[17]. Although these techniques are useful to some extent for performing repair, the outcomes of such procedures in the aesthetic region of the anterior teeth are not always optimal. In particular, for severe bone defects caused by trauma and tumors, it is difficult to achieve the desired bone augmentation using these techniques; they may not be useful for achieving precise outcomes, and no standardized procedure has been established for these techniques[19, 20]. For the management of such cases, restoration of the original physiological height and thickness of the alveolar bone as accurately as possible is a very challenging task, and it also directly determines the effect of later-stage implant restoration.

Based on the above considerations, we designed this clinical study to achieve standardized and precise bone augmentation in the aesthetic region of the anterior teeth using computer-aided design. The results obtained indicated that precise onlay bone graft design ensured optimal bone augmentation at the surgical site with high precision; strong internal fixation in the operation was performed easily, and soft tissue closure was performed without any difficulty. The resorption of bone graft observed was within the expected range. The postoperative repair effect was good. In practice, we believe that the following points need attention. (1) Choice of indications: loss of more than two adjacent teeth and a defect height exceeding 1.5 cm are necessary for application of computer-aided design and the surgical template. In case of a small defect, direct intraoperative designing is sufficient for performing bone augmentation. (2) Residual bone handling in the surgical area: considering the accuracy of the operation, if necessary, the weak bony wall should be removed and the shape of the defect area should be trimmed to achieve a close approximation with the graft bone. As expected, bone resorption in the bone-binding region was not found in our study. (3) Design of bone graft: the iliac crest is recommended as an occlusal surface of the graft bone and the cortical bone lateral to the iliac bone acts as the labial aspect of the graft bone. (4) Bone resorption: a previous study[21] demonstrated that average resorption of the labial bone is 1.24 mm, greater than 1 mm, but this can be overcome by guided bone regeneration or bone splitting and bone expansion in second-stage surgery. Moreover, if the thickness of the labial bone is increased, it will inevitably worsen the deficiency of soft tissues on the labial side, which is not conducive to closure of the surgical wound. Therefore, it is recommended to design the graft bone 1-mm thicker than needed for bone resorption. The amount of bone resorption on the palatine side is not different from the design. The average amount of vertical bone resorption is 1.09 mm, and the preoperative design is 1 mm lower than the enamel-cementum junction. The bone edge is located 2–3 mm below the enamel-cementum junction, which is the same as the physiological position[21, 22]. (5) Incision design and treatment of labial soft tissues: An incision is suggested on the alveolar ridge crest, accompanying bilateral longitudinal incisions. A transverse periosteal incision is suggested during suturing to extend the labial soft tissue. In our clinical practice, since the bone block has a precisely-designed 3D shape, only a mini-titanium plate is needed for fixation. Direct suture is feasible, without the need for soft tissue grafting, which ensures integrity of the color and shape of the pink (gingival) aesthetic region.

In summary, free bone grafting is suitable for the management of severe bone loss in the aesthetic region of the anterior teeth. However, precise preoperative design and appropriate surgical techniques are the most important factors for ensuring good outcomes. Use of computer-aided design combined with a 3D-printed template may be an effective method for achieving accurate bone augmentation in the aesthetic region of the anterior teeth.

## Data Availability

All data generated or analysed during this study are included in this published article .
